# Changes in Job Quality as People Work Beyond Pensionable Age in Sweden

**DOI:** 10.1093/geroni/igab046.1605

**Published:** 2021-12-17

**Authors:** Lawrence Sacco, Kevin Cahill, Hugo Westerlund, Loretta Platts

**Affiliations:** 1 Stockholm University, Stockholm, Stockholms Lan, Sweden; 2 Center on Aging & Work at Boston College, Chestnut Hill, Massachusetts, United States; 3 Stockholm University, Stockholm University, Stockholms Lan, Sweden; 4 Stress Research Institute, Department of Psychology, Stockholm University, Stockholm, Stockholms Lan, Sweden

## Abstract

This paper uses data from the biennial Swedish Longitudinal Occupational Survey of Health to examine changes in job quality among older workers, controlling for work intensity and employment characteristics. Job quality outcomes included job satisfaction and physical (dangerous, strenuous or unpleasant work) and psychosocial (job strain, effort-reward imbalance, work time control) working conditions. First difference estimation was used to analyze within-individual changes in job quality, as well as changes in hours, employment characteristics (shifting to a non-permanent contract, the private sector and self-employment) and health. Individuals who worked beyond pensionable age experienced statistically significant improvements in job quality, with larger improvements among those who reduced working hours and shifted from permanent to non-permanent contracts, from the public into the private sector, and from wage-and-salary to self-employment. We conclude that work beyond pensionable age is a distinctive period characterized by employment that becomes more flexible and rewarding and less stressful.

